# Creating an interactive database for nasopharyngeal carcinoma management: applying machine learning to evaluate metastasis and survival

**DOI:** 10.3389/fonc.2024.1456676

**Published:** 2024-10-07

**Authors:** Yanbo Sun, Jian Tan, Cheng Li, Di Yu, Wei Chen

**Affiliations:** Department of Otorhinolaryngology, The Central Hospital of Wuhan, Tongji Medical College, Huazhong University of Science and Technology, Wuhan, China

**Keywords:** nasopharyngeal carcinoma, distant metastasis, machine learning, random survival forest, survival prediction

## Abstract

**Objective:**

Nasopharyngeal carcinoma (NPC) patients frequently present with distant metastasis (DM), which is typically associated with poor prognosis. This study aims to develop and apply machine learning models to predict DM, overall survival (OS), and cancer-specific survival (CSS) in NPC patients to provide optimal tools for improved predictive accuracy and performance.

**Methods:**

We retrieved over 8,000 NPC patient samples with associated clinical information from the Surveillance, Epidemiology, and End Results (SEER) database. Utilizing two methods for handling missing values—imputation or deletion—we created various cohorts: DM-all, DM-slim, OS-all, OS-slim, CSS-all, and CSS-slim. Five machine learning models were deployed for the binary classification task of DM, and their performance was evaluated using the area under the curve (AUC). For the survival prediction tasks of OS and CSS, we constructed 45 combinations using nine survival machine learning algorithms. The Concordance Index (C-index), 5-year AUC, and Brier score assessed model accuracy. Patients were stratified into two risk groups for survival analysis, and the survival curves were presented.

**Results:**

This study examines the relationships between clinical factors and survival in NPC patients. The analysis, visualized through forest plots, indicates that demographic and clinical variables like gender, marital status, tumor grade, and stage significantly affect metastatic risks and survival. Specifically, factors such as advanced stages increase metastasis and survival risks, while enhanced treatments improve survival rates. In the cohort for DM prediction, results revealed that the random forest model was the most effective, with an AUC of 0.687. In contrast, when predicting overall survival (OS), the random survival forest (RSF) model consistently showed superior performance with the highest mean C-index of 0.802, a 5-year AUC of 0.857, and a Brier score of 0.167. Similarly, for cancer-specific survival (CSS) prediction, the RSF model demonstrated a mean C-index of 0.822, a 5-year AUC of 0.884, and a Brier score of 0.165. An online Shiny server was developed to allow the models to be used freely and efficiently via http://npcml.shinyapps.io/NPCpre.

**Conclusion:**

This study successfully established an online tool by machine learning models for NPC metastasis and survival prediction, providing valuable references for clinicians.

## 1 Introduction

Nasopharyngeal carcinoma (NPC), with an incidence that is steadily increasing, is among the most common cancers affecting the human head and neck (1). Although it is relatively rare globally, NPC is notably prevalent in Eastern and Southeastern Asia (2). In areas with high incidence, rates can reach 15–50 per 100,000 people (3). Epstein-Barr virus (EBV) infection is considered the primary cause of NPC, with the virus utilizing various strategies to support the immune escape during both latency and productive infection (4). Factors such as smoking, preserved foods, and air pollution also contribute to the development of NPC (5). Unlike other cancers, surgical resection is not the primary treatment option for NPC due to its inaccessible anatomical location. Instead, radiotherapy, either alone or combined with chemotherapy, is the mainstay treatment for early or non-metastatic NPC (6). Treatment protocols differ significantly between patients with non-metastatic NPC and those with recurrent or metastatic disease. For metastatic NPC, anti-PD-1 monoclonal antibodies have effectively improved survival. A recent meta-analysis found that the overall response rate of metastatic NPC was 73% when anti-PD-1 antibodies were combined with Gemcitabine and Cisplatin (7). These treatment variations between non-metastatic and metastatic NPC underscore the importance of accurate predictions for NPC metastasis. Furthermore, accurate predictions of OS and CSS help customize treatments and improve patient prognoses. Early and aggressive interventions for individuals identified as high-risk for metastasis or poor survival can prolong life and minimize complications.

Recently, machine learning (ML) has significantly transformed the field of survival prediction due to its ability to process these non-linear interactions within data. For instance, a stacked predictive ML model demonstrated an 85.9% accuracy in stratifying NPC patients into survival probability groups (8). In another study, survival support vector machines and random survival forests were used to predict NPC survival outcomes, with C-index values of 0.785 and 0.729, respectively (9). These studies demonstrate that ML algorithms can accurately predict survival outcomes for patients with NPC. Despite these advancements, there remains a clear gap in the availability of an ML-based online tool for predicting metastasis and survival in NPC. Our study seeks to address this gap by developing online tools that apply popular ML algorithms for NPC metastasis and survival prediction, aiming to advance treatment strategies and improve patient care through more precise prognostic assessments.

Our research employed clinical variables to develop 5 binary machine learning classifiers for predicting metastasis and 45 survival machine learning classifiers to predict OS and CSS. Evaluations on testing datasets have shown that these classifiers can accurately predict outcomes. To enhance accessibility and usability, we created an online web server. This platform enables clinicians and patients to easily access these predictive tools, facilitating better decision-making in treatment strategies and improving patient outcomes through timely and personalized interventions.

## 2 Materials and methods

### 2.1 Data selection

We conducted a retrospective study on NPC patients using data from the SEER program. The SEER dataset, covering the period from 2000 to 2021 and including records from 17 registries, comprises over 9 million tumor records. This dataset mirrors the overall demographic composition, cancer incidence, and mortality rates across the nation. Access to the SEER database was granted following a formal application process, and the data was retrieved using SEER*Stat software. Since SEER is a publicly available database with de-identified data, institutional review board approval and formal patient consent were not required for this study. This study adhered to the World Medical Association’s Declaration of Helsinki for Ethical Human Research.

The inclusion criteria for NPC samples in the SEER database included the following: (1) The primary site disease code for selecting NPC is C11 (10). (2) The selected histology subtypes in patients included keratinizing squamous cell carcinoma (KSCC), differentiated non-keratinizing carcinoma (DNKC), undifferentiated non-keratinizing carcinoma (UNKC), and Others. (3) NPC was the first and only primary malignancy. (4) Additionally, patients’ survival time should be over 0 months. (5) We excluded patients with missing or unknown survival data and those whose reporting source was autopsy or death certificate only.

### 2.2 Variables of interest

This study organized, categorized, and preprocessed the downloaded clinical baseline data. The demographic variables examined included age (continuous variable), sex (male or female), race (Hispanic, white, black, American Indian or Alaska Native (AA), and Asian or Pacific Islander (AP)), and marital status (partnered: married or domestic partner; previously partnered: divorced, separated, widowed; and single). The clinical variables include histological subtypes, tumor site, tumor grade, Tumor-node-metastasis (TNM) staging system, overall stage, and tumor size. Histological subtypes were classified into DNKC, KSCC, UNKC, and Others. Tumor sites were detailed as anterior wall, lateral wall, overlapping lesion, posterior wall, superior wall, and unspecified sites. Tumors were graded as Grade I, Grade II, Grade III, and Grade IV. TNM staging system was delineated as T1 to T4, N0 to N3, and M0 to M1. Tumor size (continuous variable) was also included. Treatment Variables included Surgery for the primary site (SurgPS), Surgery for lymph nodes (SurgLN), Chemotherapy, Radiotherapy, and Time-to-treatment. SurgPS was categorized as No Surgery, Local Excision, Pharyngectomy, and unspecified Surgery. SurgLN included Biopsy, Lymph Nodes Removed, and None. Chemotherapy was recorded as No/Unknown and Yes, while radiotherapy was categorized as Beam Radiation, Other Radiation, and No/Unknown. Time-to-treatment (time from diagnosis to treatment in days) was quantified as Timely (<30 days), Intermediate (30-90 days), and Long (>90 days).

The study’s primary outcomes focused on Distant Metastasis (DM), Overall Survival (OS), and Cancer-Specific Survival (CSS) among patients with NPC. DM was defined as M1 in the TNM staging system. OS was defined as the duration from diagnosis to death from any cause, while CSS was defined as the time from diagnosis until death directly attributable to NPC. In the DM cohort, demographic variables included age, gender, race, and marital status, while clinical variables included histology subtype, site, grade, tumor size, T stage, and N stage. For the OS and CSS cohorts, demographic variables included age, gender, race, and marital status, and clinical variables included histology subtype, site, grade, tumor size, T stage, N stage, and M stage. Additionally, treatment variables included primary surgery (SurgPS), lymph node surgery (SurgLN), chemotherapy, radiotherapy, and time-to-treatment.

### 2.3 Cohort separation and data preparation

Managing missing values in datasets is a widely debated topic within data science. Our research used the K-Nearest Neighbors method to impute missing values. Alternatively, another strategy involves removing variables with more than 30% missing values and excluding samples with any missing values. These methodologies led to the formation of two distinct sets of cohorts for each group. For the DM group, we created the DM-all cohort, where missing values were imputed, and the DM-slim cohort, which consists only of samples with complete data. For the OS group, we created the OS-all cohort with imputed missing values and the OS-slim cohort with only complete data. Similarly, the CSS-all cohort includes imputed data for the CSS group, while the CSS-slim cohort comprises only fully observed data. These approaches allow for comprehensive data analysis while catering to different data integrity preferences. Subsequently, these cohorts were randomly divided into training (70%) and testing (30%) subsets.

### 2.4 Models for binary classification of DM status

Due to the imbalance ratio of distant metastasis vs. non-distant metastasis, we employed a method to balance the dataset. This study applied the Synthetic Minority Oversampling Technique (SMOTE) to the metastasis samples in the training sets. SMOTE offers four key advantages over other techniques. (1) It has been widely validated and is known for effectively addressing class imbalance in medical datasets, including those involving survival analysis and binary classification problems (11). (2) A significant benefit of SMOTE is its simplicity and transparency, as it generates synthetic samples for the minority class based on the k-Nearest Neighbors algorithm. (3) SMOTE provides a good balance between efficiency and performance. While generative adversarial networks show advantages for handling imbalanced datasets (12), SMOTE is more straightforward to implement. (4) SMOTE enhances the representation of minority classes without significantly altering the overall data distribution, thereby helping to maintain model generalizability. In this study, SMOTE was only applied to the training set.

In this study, we constructed models using five machine learning algorithms: Gradient Boosting Machine (GBM), Decision Tree (Tree), K-Nearest Neighbors (KNN), Random Forest (RF), and Generalized Linear Model (GLM). We trained five models using the training sets from DM-all and DM-slim cohorts and then tested the models on testing sets from DM-all and DM-slim cohorts. To determine the ideal model parameters, we used a random hyperparameter search and average AUC values under 5-fold cross-validation for every methodology. Additionally, we used plots to assess the relative importance values of clinical variables using the random forest model.

### 2.5 Models for survival classification of OS and CSS

In analyzing right-censored survival data, various machine learning methods are employed to handle datasets effectively. The “rfSRC” model utilizes the Random Survival Forest methodology, constructing an ensemble of survival trees that enhance prediction accuracy through a collective voting mechanism (13). The “CoxPH” model applies the traditional Cox Proportional Hazards framework to efficiently estimate hazard ratios without defining a baseline hazard (14). The “CoxBoost” model extends this approach by incorporating boosting techniques to improve the performance of the Cox proportional hazards model. The “GBM” method employs Gradient Boosting Machine principles, adeptly correcting prediction errors sequentially with an assembly of decision trees, effectively managing nonlinear relationships within censored survival data. Additionally, “superPC” utilizes principal component analysis optimized for survival outcomes, concentrating on the most significant predictors. The “stepCox” method streamlines the variable selection process within the Cox model, enhancing accessibility and ease of use. Regularization techniques such as “Lasso”, “Ridge”, and “Enet” are implemented to prevent overfitting and improve model accuracy by imposing penalties on complex models, thus enhancing their generalization across different datasets. After building individual models for each survival machine learning algorithm, we performed model combinations by integrating their outputs and calculating the mean of two models. For example, if the predicted values from the rfSRC and CoxPH models are 0.6 and 0.8, respectively, the combined prediction from the rfSRC_plus_CoxPH model would be 0.7. This combination approach resulted in a total of 45 machine learning models, consisting of 9 individual models and 36 combined models.

### 2.6 Evaluation of survival machine learning models

The OS-all, OS-slim, CSS-all, and CSS-slim cohorts were randomly separated into training (70%) and testing (30%) sets. We followed a comprehensive machine learning workflow (MLW) for training and testing: 45 survival machine learning models were trained on the training set and validated on the testing set. We conducted cross-validation within the training set to reduce potential bias from the random split. Specifically, we divided the training set into five folds, where in each iteration, one fold served as the validation set while the remaining four folds were used for training. This MLW process ensured that all samples in the dataset were used for testing. The machine learning combinations were validated by assessing the performance of discrimination and calibration between the five folds of training and validation sets. The C-index, the AUC at five years, and the Brier score were used to assess the models’ discriminating ability. A higher C-index and AUC prove the model’s predictions and actual events agree. Conversely, a lower Brier score indicates higher accuracy in survival data. Finally, the importance values of clinical variables in the models were plotted.

### 2.7 Survival differences of different risk groups

The random survival forest model was used for every patient in the testing set to generate the overall risk score. Patients were divided into low- and high-risk categories using the median risk score as a guide. Using log-rank tests and Kaplan-Meier survival curves, the OS and CSS of patients in risk categories were plotted and contrasted.

### 2.8 Online web server by Shiny

To make the predictive models accessible, we developed an online web server using the Shiny package in R. We formatted the data to meet Shiny’s input and output requirements. Machine learning models were connected to provide real-time predictions. A simple interface was created with fields for clinical variables and results for predicted DM risk scores and survival. The online web server was hosted on a cloud server to ensure easy access and scalability. Our online platform allows medical professionals and researchers to offer patients with NPC more tailored treatment plans and risk evaluations.

## 3 Results

### 3.1 The association of variables with outcomes

**Figure 1:**
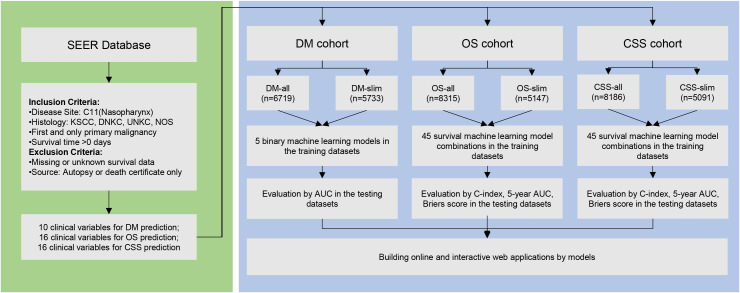
Flowchart of patient selection and model construction in this study.

**Figure 2:**
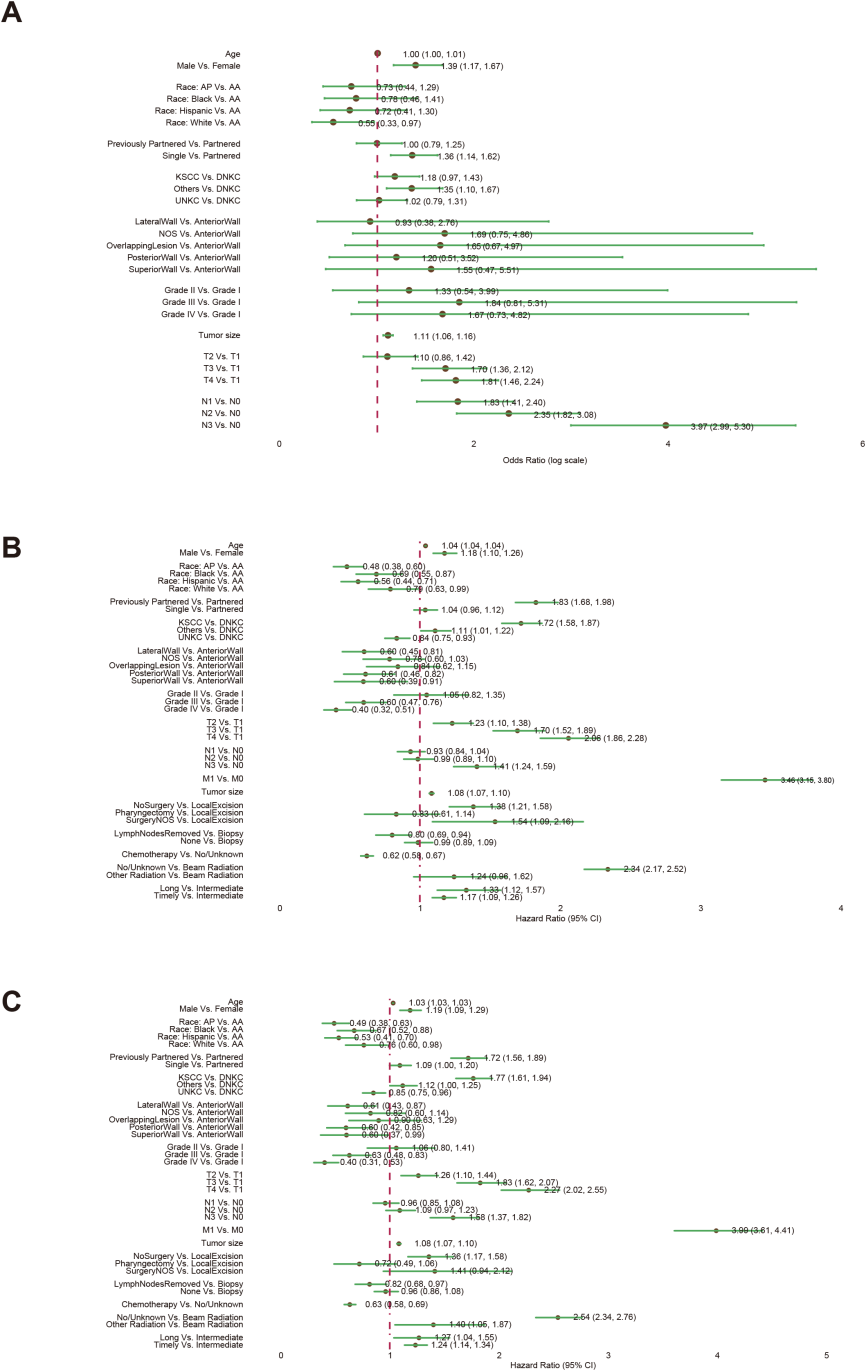
The associations of clinical variables with distant metastasis (DM), overall survival (OS), and cancer-specific survival (CSS). Logistic regression analysis was used for the DM analysis **(A)**. Univariate Cox regression analysis was used for the OS **(B)** and CSS **(C)** analyses. Abbreviations: American Indian or Alaska Native (AA); Asian or Pacific Islander (AP); Keratinizing Squamous Cell Carcinoma (KSCC), Differentiated Non-Keratinizing Carcinoma (DNKC), Undifferentiated Non-Keratinizing Carcinoma (UNKC).

After screening, our study incorporated 6,709 nasopharyngeal carcinoma (NPC) samples into the Disease Metastasis (DM) cohort, characterized by 10 unique clinical variables. Additionally, we analyzed 8,315 NPC samples in the Overall Survival (OS) cohort and 8,186 samples in the cancer-specific survival (CSS) cohort, each with 16 distinct clinical variables (**Figure 1**). We used a forest plot to visualize the odds ratios (ORs) of various variables associated with the metastasis of NPC (**Figure 2A**). Notably, being male, single, and having a larger tumor size are associated with increased odds of metastasis. Additionally, advanced T stages (T3, T4) and N stages (N1, N2, N3) significantly elevate the odds of metastasis. Race also plays a role, with White patients showing lower odds compared to American Indian or Alaska Native (AA). These findings highlight the influence of demographic, clinical, and tumor-specific variables on the metastatic progression of NPC.

The forest plot from the univariate Cox proportional hazards model highlights the influence of various factors on OS (**Figure 2B**). Key findings reveal that older age and male gender are linked to modest increases in hazard ratios. Specific racial groups such as Asian or Pacific Islanders (AP), Blacks, and Hispanics exhibit significantly better survival rates compared to AA. Tumors located in the Lateral Wall, Posterior Wall, and Superior Wall demonstrate improved survival compared to those in the Anterior Wall. Contrary to most tumor types, higher tumor grades are associated with enhanced survival. Advanced TNM staging and larger tumor sizes, particularly when involving metastasis, significantly elevate the risks. Treatment strategies also play a crucial role: extensive surgeries and chemotherapy boost survival, whereas time-to-treatment correlates with poorer outcomes. Specifically, beam radiation markedly improves survival. Similar associations of clinical variables with the survival outcome of Cancer-Specific Survival (CSS) are observed through a forest plot (**Figure 2C**).

### 3.2 Data preprocessing and cohort separation

Our approach used the KNN method to impute missing values, forming the Distant Metastasis (DM), Overall Survival (OS), and Cancer-Specific Survival (CSS) cohorts, named DM-all, OS-all, and CSS-all, respectively. Alternatively, we removed samples with any missing values, thus creating the DM-slim, OS-slim, and CSS-slim cohorts. Comprehensive baseline information for these cohorts can be found in **Supplementary Tables 1**–**Supplementary Tables 6**. Then, we divided the data set into the training and testing sets in a 7:3 ratio using random sampling. For the predictive modeling phase, the DM-all cohort included 4,699 patients in the training and 2,010 in the testing set, with 10 available variables. The DM-slim cohort comprised 4,014 and 1,719 patients in the training and testing sets, respectively, with 8 variables. In the OS-all cohort, 5,823 patients were allocated to the training and 2,492 to the testing set with 16 variables. The OS-slim cohort included 3,605 patients in the training set and 1,542 in the testing set, with 14 clinical variables. Similarly, the CSS-all cohort had 5,732 patients in the training set and 2,454 in the testing set with 16 variables. In comparison, the CSS-slim cohort included 3,565 patients in the training set and 1,526 in the testing set with 14 clinical variables. These allocations are designed to ensure robust training and validation phases, enhancing the accuracy and reliability of our predictive models across these specific cohorts.

### 3.3 Model performance for predicting DM

**Figure 3:**
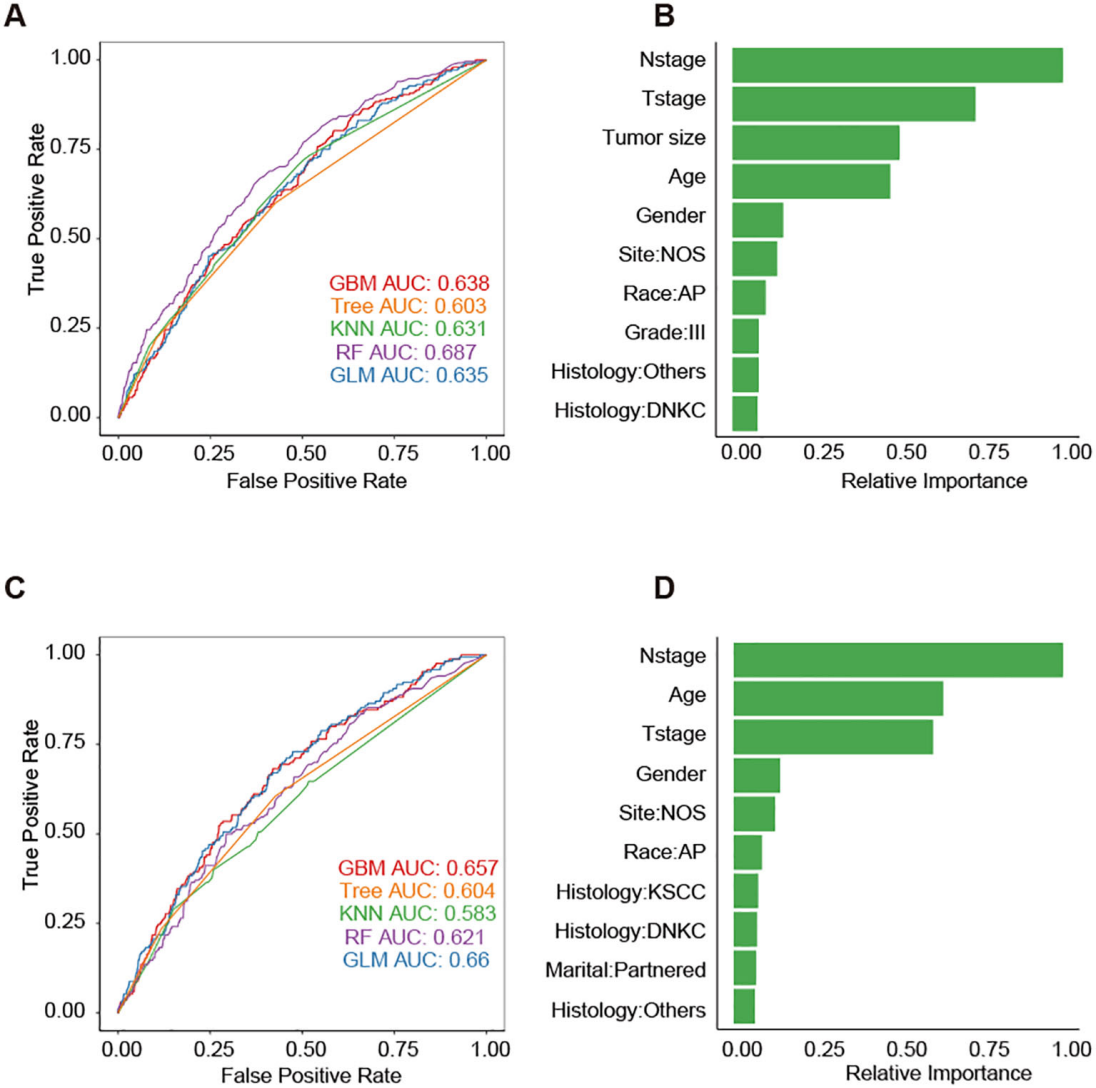
Five machine learning models for predicting the DM of NPC. **(A)** The AUC values of models in the testing set of the DM-all cohort. **(B)** The relative importance values clinical variables in the random forest model from the testing set of the DM-all cohort. **(C)** The AUC values of models in the testing set of DM-slim cohort. **(D)** The relative importance values clinical variables in the random forest model from the testing set of the DM-slim cohort. Abbreviations: gradient boosting machine (GBM); decision tree (Tree); k-nearest neighbors (KNN); random forest (RF); generalized linear model (GLM).

To develop predictive models for DM, we trained five different machine learning algorithms on the training set of the DM-all cohort. We evaluated their performance on the corresponding testing set. Parameter tuning was conducted using five-fold cross-validation within the training set, and the optimal models were subsequently saved. The evaluation of model efficacy, based on AUC scores, indicated that the random forest algorithm achieved the highest score of 0.687 (**Figure 3A**). Analysis of feature importance in the random forest model revealed that N stage, T stage, and tumor size were the most influential variables in predicting DM (**Figure 3B**). Similarly, we trained and tested the machine learning models for the DM-slim cohort. In this cohort, the generalized linear model (GLM) yielded the highest AUC value of 0.66 (**Figure 3C**). Clinical variables such as N stage, age, and T stage were identified as having the most significant impact on DM prediction in the DM-slim cohort (**Figure 3D**).

### 3.4 Model performance for predicting OS

The study’s OS-all dataset comprised 8,315 NPC samples with 16 clinical variables. These were randomly divided into a training set containing 5,823 samples and a testing set containing 2,492. We adopted a comprehensive machine learning workflow (MLW), training 45 survival machine learning models on the training set and validating their performance on the testing set using three key metrics: the C-index, AUC, and IBS scores. To ensure robustness and reduce bias from the random division, we performed cross-validation within the training set by dividing it into five folds. In each iteration, one fold was the validation set, and the remaining four were used for model training. This procedure guaranteed that every sample in the dataset was utilized for testing.

**Figure 4:**
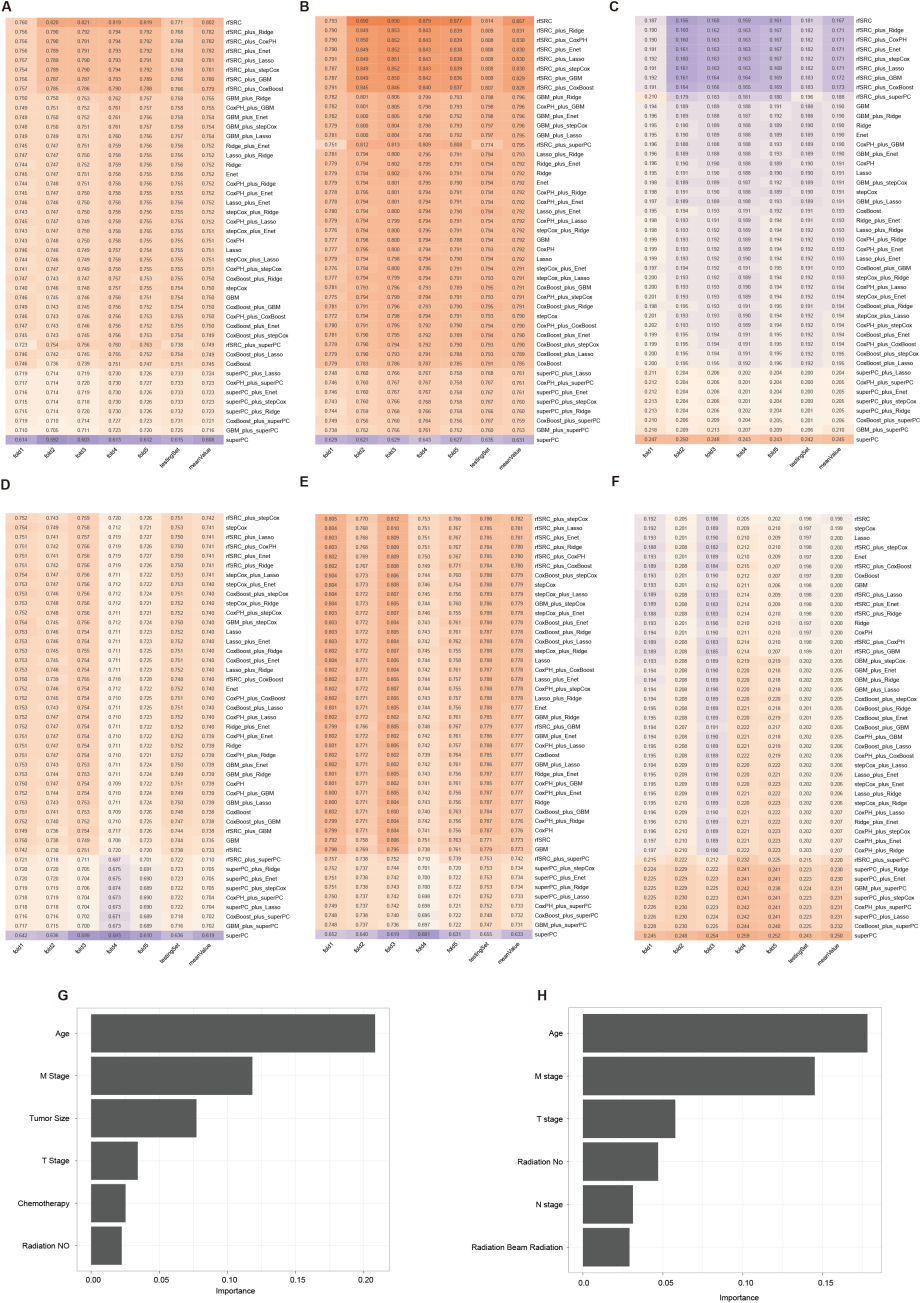
The 45 machine learning combinations for predicting the OS in the OS-all and OS-slim cohorts. The C-index **(A)**, 5-year AUC **(B)**, and Brier score **(C)** of models in the OS-all cohort. The C-index **(D)**, 5-year AUC **(E)**, and Brier score **(F)** of models in the OS-slim cohort. The importance values of clinical variables in the random survival forest model from the OS-all **(G)** and OS-slim **(H)** cohort.

**Figure 5:**
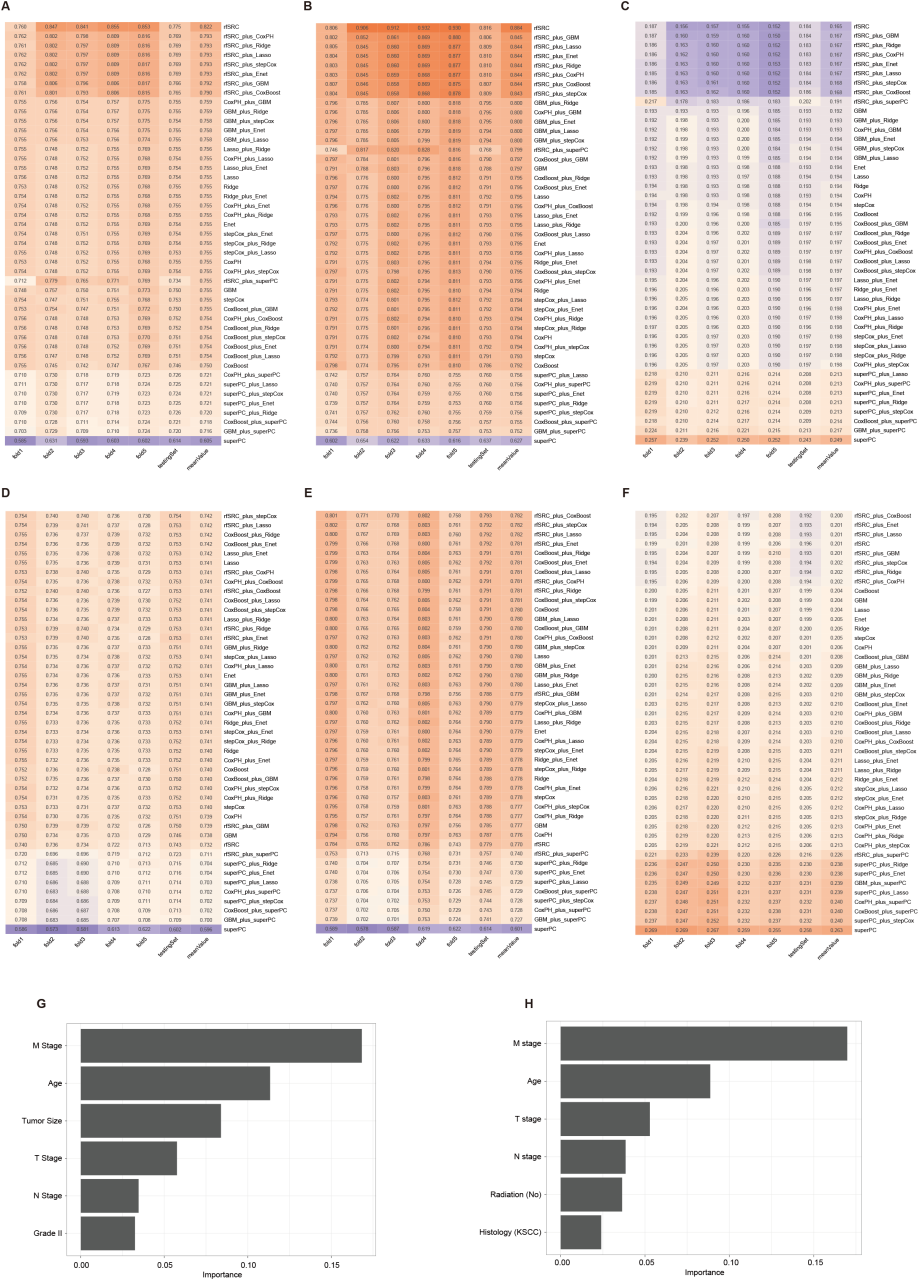
The 45 machine learning combinations for predicting the CSS in the CSS-all and CSS-slim cohorts. The C-index **(A)**, 5-year AUC **(B)**, and Brier score **(C)** of models in the CSS-all cohort. The C-index **(D)**, 5-year AUC **(E)**, and Brier score **(F)** of models in the CSS-slim cohort. The importance values of clinical variables in the random survival forest model from the CSS-all **(G)** and CSS-slim **(H)** cohort.

Among all model combinations in the OS-all cohort, the rfSRC model achieved the highest average C-index value of 0.802, with individual fold results ranging from 0.760 to 0.821 and testing at 0.771 (**Figure 4A**). The rfSRC model also excelled in 5-year AUC (**Figure 4B**) and Brier score (**Figure 4C**), recording 0.857 and 0.167 respectively. For the OS-slim cohort, which included 3,605 patients in the training set and 1,542 in the validation set with 14 clinical variables, the rfSRC_plus_stepCox combination displayed the highest mean C-index (**Figure 4D**) and 5-year AUC (**Figure 4E**) values of 0.742 and 0.782, respectively. In terms of the Brier score (**Figure 4F**), the rfSRC model stood out by showing the lowest mean value of 0.198, indicating its high predictive accuracy. The rfSRC model calculated each variable’s importance (VIMP) using the VIMP method, which helped rank the variables by importance. The six most critical variables identified in predicting OS-all were Age, M stage, tumor size, T stage, chemotherapy, and radiation (No) (**Figure 4G**). The six most critical variables identified in predicting OS-slim were Age, M stage, T stage, radiation (No), N stage, and radiation (beam radiation) (**Figure 4H**).

### 3.5 Model performance for predicting CSS

The CSS-all dataset included 5732 patients in the training set and 2454 in the validation set, with 16 clinical variables. Among all 45 machine learning combinations, rfSRC performed the best, showing the highest mean C-index (0.822), the highest 5-year AUC (0.884), and the lowest Brier score (0.165) as illustrated in **Figures 5A-C**, respectively. The CSS-slim dataset included 3565 patients in the training set and 1526 in the validation set, with 14 clinical variables. rfSRC-related models performed as the best model in this cohort, showing the highest mean C-index (0.742), the highest 5-year AUC (0.782), and the lowest Brier score (0.200), as shown in **Figures 5D-F**. For the CSS-all dataset, the top three most important variables in predicting outcomes were M stage, Age, and Tumor size, as shown in **Figure 5G**. For the CSS-slim dataset, the top three most important variables were the M stage, Age, and T stage, as shown in **Figure 5H**.

### 3.6 Survival analysis of subgroup analysis based on risk stratification

**Figure 6:**
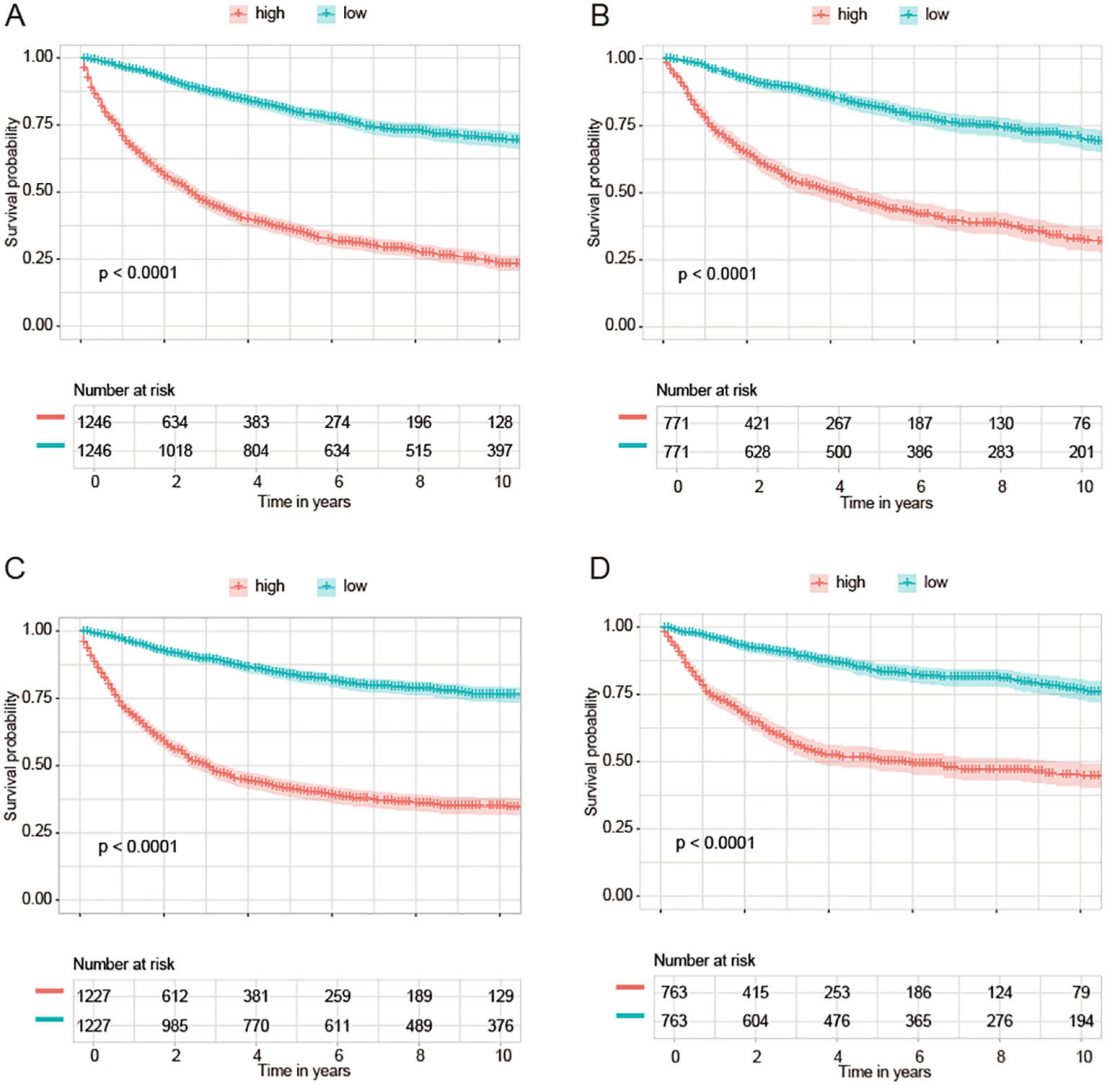
KM curves of survival are based on the machine learning-based risk score. KM curves of groups of OS from OS-all cohort **(A)** and OS-slim cohort **(B)**. KM curves of groups of CSS from CSS-all cohort **(C)** and CSS-slim cohort **(D)**.

We divided NPC samples into subgroups based on the median predicted risk of death as determined by the machine learning model (rfSRC). This division was done to highlight the benefits of risk stratification. In conducting survival analyses of these different risk subgroups, we observed distinct prognostic outcomes: individuals in the high-risk group exhibited poorer prognoses, whereas those in the low-risk group demonstrated better prognoses. This pattern was consistently observed across various datasets: in OS-all (**Figure 6A**), OS-slim (**Figure 6B**), CSS-all (**Figure 6C**), and CSS-slim (**Figure 6D**).

### 3.7 Development of an online distant metastasis and survival estimate calculator

**Figure 7:**
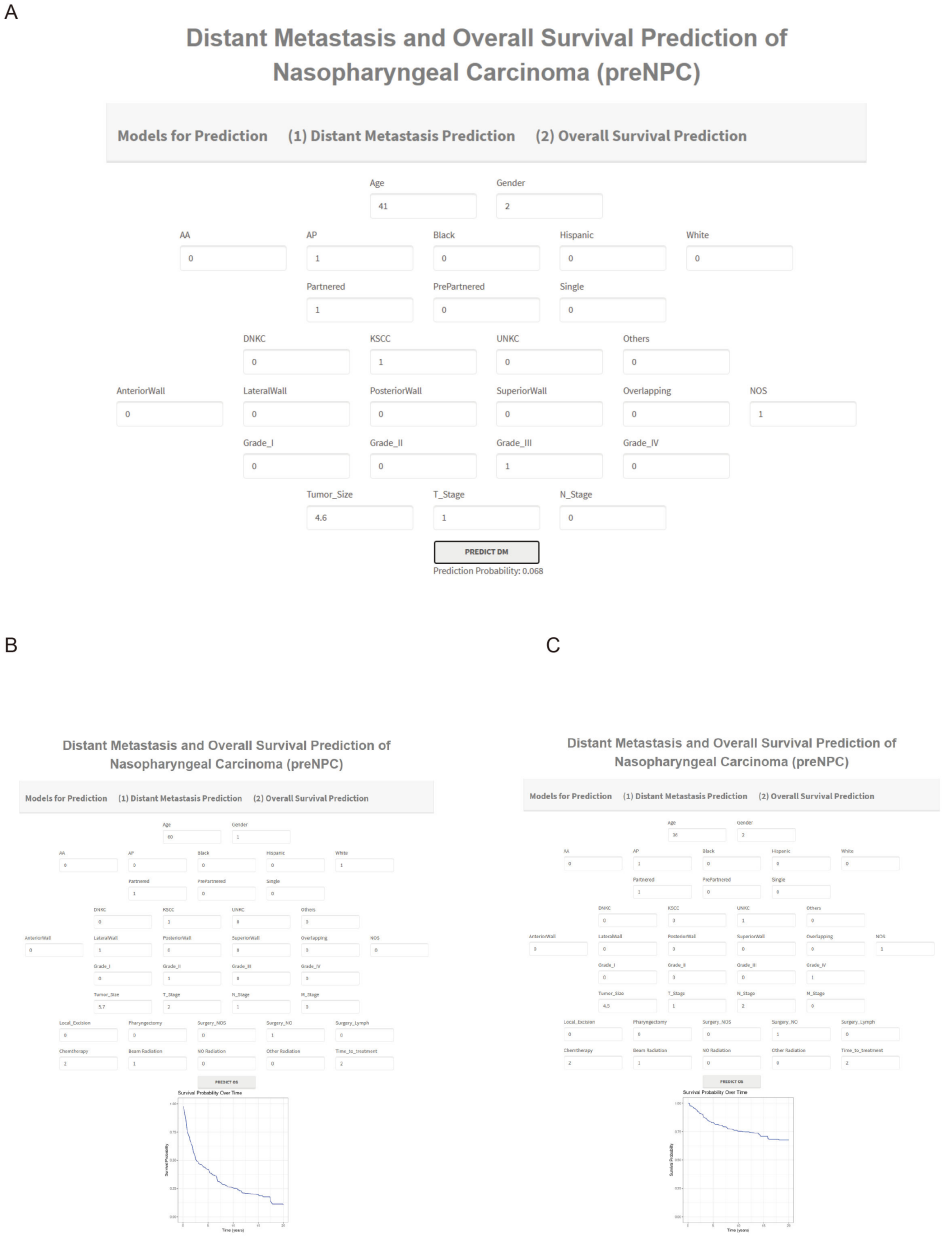
Examples of Using NPCpre to Predict DM **(A)** and OS **(B, C)**. Parameter Descriptions for Predicting DM **(A)**. Age in years; Gender: Male (1), Female (2); Race of Patient: Hispanic, American Indian or Alaska Native (AA), Asian or Pacific Islander (AP), Black, and White; Marital Status: Partnered, Previously Partnered, Single; Histology Subtype: Keratinizing Squamous Cell Carcinoma (KSCC), Differentiated Non-Keratinizing Carcinoma (DNKC), Undifferentiated Non-Keratinizing Carcinoma (UNKC), Others; Tumor Locations: Anterior wall, lateral wall, overlapping lesion, posterior wall, superior wall, Not Otherwise Specified (NOS); Grade of Patient: I to IV; Tumor Size in centimeters; T Stages: 1 to 4; N Stages: 0 to 3. Additional Parameters for Predicting OS **(B, C)**. M Stage: 0 to 1; Surgery for the Primary Site (SurgPS): Local Excision, Pharyngectomy, Surgery Not Otherwise Specified, No Surgery; Surgery for Lymph Nodes (SurgLN): 0 for None, 1 for Biopsy, 2 for Lymph Nodes Removed; Chemotherapy: 1 for No/Unknown, 2 for Yes; Radiation: Beam Radiation, No Radiation, Other Radiation Types; Time-to-treatment: 1 for Timely (<30 days), 2 for Intermediate (30-90 days), 3 for Long (>90 days). For example, plot **(C)** features a 36-year-old female, race AP, partnered, with UNKC subtype, tumor location NOS, Grade IV, tumor size 4.5 cm, T1, N2, M0, did not receive SurgPS and SurgLN, received Chemotherapy and beam radiation. The days between diagnosis and treatment were 30-90 days.

To facilitate its use by clinicians, we developed an online tool using the ‘shiny’ package. This web server is available at http://npcml.shinyapps.io/NPCpre. Upon entering the required clinical parameters, the tool displays predicted rates of distant metastasis (DM) and survival curves, illustrating changes in survival rates over time. **Figures 7A-C** demonstrates the use of this tool for predicting DM and overall survival (OS). For instance, **Figure 7A** presents a case from the SEER database of a real-world NPC patient with ID 897528, who belongs to the testing cohort of our study and is therefore not used in model training. The NPCpre web server predicts that this patient’s DM probability is 0.068, and actual follow-up data confirmed that the patient did not develop DM. Similarly, to showcase the prediction capability of our tool regarding OS, we randomly selected two NPC patients (62896440 and 9081770) from the testing cohort. **Figure 7B** illustrates that the survival rate for patient 62896440 was predicted to drop significantly from 1 to 0.5 within the first 2.5 years, aligning with actual follow-up data indicating the patient’s death approximately 1 year after the treatment. **Figure 7C** reveals that patient 9081770 maintained a survival rate higher than 0.5 for over 20 years, corroborated by follow-up data showing this patient is still alive after over 13 years. The consistency between predictions from NPCpre and actual follow-up data indicated our model’s and webserver’s robustness. This tool provides a valuable resource, allowing physicians and patients to individually and visually evaluate the survival probabilities of each patient by common clinical variables.

## 4 Discussion

Accurately predicting distant metastasis (DM), overall survival (OS), and cancer-specific survival (CSS) in patients with nasopharyngeal carcinoma (NPC) is crucial for advancing research in this field. These predictive capabilities allow for the customization of treatments, planning of follow-ups, and improvement of patient prognosis, particularly given the treatment variations between non-metastatic and metastatic NPC. The current study demonstrates the effectiveness of machine learning (ML) models in accurately predicting DM, OS, and CSS in NPC patients. To the best of our knowledge, this might be the first online tool that utilizes ML models for assessing metastasis and survival outcomes in NPC. This study shows the potential of integrating artificial intelligence into clinical prognostics, offering a more accessible and potentially precise method for healthcare professionals to evaluate disease progression and survival rates.

DM, the primary cause of treatment failure in advanced NPC, remains a significant challenge. Predicting DM is essential for guiding individualized treatment plans for NPC patients. Traditional methods have used genomic and clinical features for this purpose. For instance, a nomogram based on immune markers (PD-L1+ CD163+, CXCR5, CD117) showed predictive performance with a C-index of 0.729 in the validation cohort (15). Another study’s nomogram achieved a C-index of 0.718 in the validation cohort (16). Given the superior performance of machine learning models over traditional nomograms, developing machine learning models for predicting DM holds great promise for enhancing predictive accuracy. Besides, these studies usually focus on predicting DM after chemotherapy. However, the prediction of DM at the primary diagnosis should also be included. In the current study, we used machine learning models to predict the DM at primary diagnosis instead of nomograms to predict DM after treatment. We selected five machine learning algorithms to predict DM, a binary classification task (non-metastasis or metastasis). Despite adopting these machine learning models, the number of variables in the SEER database for predicting DM at primary diagnosis is limited. Our study used age, gender, race, marital status, histology type, tumor site, tumor grade, tumor size, T stage, and N stage.

The prognosis of cancer is influenced by multiple factors, making traditional linear statistical models potentially unreliable for predicting survival. In many studies, nomograms using conventional models are prevalent tools for predicting the survival of NPC patients. One research group developed a nomogram using eight clinical variables to predict overall survival (OS) in patients aged 18 to 59 with NPC, reporting a C-index of 0.69 and a 5-year AUC of 0.729 (17). Another group provided interactive nomograms for predicting OS in NPC, achieving a 5-year AUC of 0.74 and a C-index of 0.70 in their testing cohorts (18). A different nomogram, constructed using four independent risk indicators (histology, radiation therapy, chemotherapy, and metastatic status), reported AUC values of 0.733 for 3-year cancer-specific survival (CSS) and 0.719 for 5-year CSS (19). Other research groups have also constructed nomograms for OS prediction in NPC (20, 21). However, the main drawback of non-machine learning models is their suboptimal performance, with C-index and AUC values typically below 0.80.

To develop more advanced prediction models with C-index and AUC values exceeding 0.80, researchers have begun adopting several machine learning (ML) algorithms to predict NPC prognosis. A review summarized various publications employing ML for NPC management (22). Using 123 MRI images, one study developed a radiomics nomogram by integrating a radiomics signature, achieving a C-index of 0.863 for personalized risk stratification (23). Another study applied neural networks to analyze pathological microscopic features, reaching a C-index of 0.723 (24). Additionally, some research focuses on using clinical factors, which are easier to implement in clinical practice. One research group developed a stacked predictive ML model showing an accuracy of 85.9%. At the same time, the XGBoost algorithm achieved 84.5% accuracy after the training and testing phases (8). Another study used a survival support vector machine and random survival forest models to predict NPC survival, obtaining a C-index of 0.785 for the survival-SVM model and 0.729 for the RSF model (9). However, unlike nomogram-based studies, which are easily accessible to clinicians, these ML-based studies have not provided user-friendly tools, such as web applications, for broader clinical use.

We tested different combinations of nine machine learning algorithms (rfSRC, CoxPH, CoxBoost, GBM, superPC, stepCox, Lasso, Ridge, and Enet) to identify the optimal model combinations for survival prediction in NPC. The performance results showed that the random forest survival (rfSRC) model and its combinations had superior accuracy in predicting survival outcomes. For predicting overall survival (OS), the best model achieved a C-index of 0.802, a 5-year AUC of 0.857, and a Brier score of 0.167. For predicting cancer-specific survival (CSS), the best model achieved a C-index of 0.822, a 5-year AUC of 0.884, and a Brier score of 0.165. These results outperform the models discussed in the previous paragraph, either in terms of available variables or overall performance. Furthermore, our models are publicly available for easy use by clinicians and patients, as we have deployed them on an online web server.

Based on the median predicted risk of death determined by the rfSRC model, we found that individuals in the high-risk group exhibited poorer prognoses. In contrast, those in the low-risk group demonstrated better outcomes. However, there are limitations to interpreting the Kaplan-Meier curves. One limitation is that Kaplan-Meier curves are based on categorical groupings (e.g., high-risk vs. low-risk) and may not fully capture the continuous nature of risk scores provided by the model. This categorization can lead to simplistic information about individual risk levels. Additionally, Kaplan-Meier curves do not account for competing risks, such as death from causes other than NPC, which could affect the interpretation of survival probabilities.

Data preprocessing, including handling missing values, is crucial in constructing machine learning models. Our study employed two distinct strategies for handling missing data: imputation and deletion. We utilized the KNN method to estimate missing values for the imputation strategy. On the other hand, our deletion strategy involved removing any variables with more than 30% missing data and excluding any samples with missing values. This approach resulted in a reduced dataset size. Specifically, in the OS-all cohort, the data comprised 5,823 patients in the training and 2,492 in the testing set, with 16 clinical variables maintained. Conversely, the OS-slim cohort, formed under the deletion strategy, included 3,605 patients in the training set and 1,542 in the testing set, with only 14 clinical variables. To compare their efficacy, we independently constructed models on both the imputation-generated and deletion-generated cohorts. The evaluation of model performance revealed that the imputation-based models consistently outperformed those generated from the deletion strategy. This superior performance can be attributed to the more significant number of variables and samples retained in the imputation approach, which are crucial for enhancing the predictive accuracy of machine learning models.

In this study, we developed machine learning models to predict distant metastasis, overall survival, and cancer-specific survival in patients with NPC. These models enhance patient stratification and inform clinical decision-making by allowing healthcare professionals to personalize treatment strategies based on metastatic or survival status. Patients predicted to have metastatic disease or worse survival outcomes may receive more aggressive systemic treatments like combination chemotherapy, targeted therapy, or immunotherapy. Conversely, those predicted not to have metastatic disease can focus on local treatments such as radiotherapy or concurrent chemoradiotherapy. By accurately identifying metastatic and survival status, clinicians can select treatments to optimize outcomes and minimize unnecessary toxicity. The accessibility of our models via an online Shiny server facilitates their integration into clinical practice. This represents a significant step toward improving patient outcomes through personalized, data-driven care.

Several limitations need to be addressed. Firstly, the SEER database provides limited information on tumor genetic profiles and biomolecular markers, essential for accurately assessing overall survival (OS) and cancer-specific survival (CSS) outcomes. For instance, incorporating genetic and biomolecular markers could improve predictive accuracy and offer deeper insights. Secondly, while metastasis information in the SEER database is recorded at initial diagnosis, metastasis data from follow-up in non-metastatic patients would be more valuable for this group. Thirdly, we lacked an external dataset with a larger sample size to test the generalization capability of our optimal model, and acquiring new data may be necessary for further validation.

## 5 Conclusion

In conclusion, we have established an online web tool using machine learning models that incorporate clinical features to predict metastasis and survival in NPC patients. This tool aims to enhance decision-making in treatment strategies and improve patient outcomes through timely and personalized interventions.

## Data Availability

The original contributions presented in the study are included in the article/**Supplementary Material**, further inquiries can be directed to the corresponding author/s.
