# Supplementary material for: Creating an interactive database for nasopharyngeal carcinoma management: applying machine learning to evaluate metastasis and survival
Source: Front Oncol. 2024 Oct 7;14:1456676. doi: 10.3389/fonc.2024.1456676 (PMC11491431; doi:10.3389/fonc.2024.1456676)
Supplement: Supplementary file 1 [file Table1.docx]

|  | **training** | **testing** | **p-value** |
| --- | --- | --- | --- |
|  | ***n=4699*** | ***n=2010*** |  |
| Metastasis: |  |  | 0.112 |
| No | 4184 (89.0%) | 1762 (87.7%) |  |
| Yes | 515 (11.0%) | 248 (12.3%) |  |
| Age | 53.6 (15.2) | 53.5 (14.9) | 0.760 |
| Gender: |  |  | 0.558 |
| Female | 1318 (28.0%) | 549 (27.3%) |  |
| Male | 3381 (72.0%) | 1461 (72.7%) |  |
| Race: |  |  | 0.537 |
| AA | 81 (1.72%) | 25 (1.24%) |  |
| AP | 2044 (43.5%) | 893 (44.4%) |  |
| Black | 535 (11.4%) | 237 (11.8%) |  |
| Hispanic | 440 (9.36%) | 176 (8.76%) |  |
| White | 1599 (34.0%) | 679 (33.8%) |  |
| Marital: |  |  | 0.424 |
| Partnered | 2915 (62.0%) | 1279 (63.6%) |  |
| Previously Partnered | 662 (14.1%) | 278 (13.8%) |  |
| Single | 1122 (23.9%) | 453 (22.5%) |  |
| Histology: |  |  | 0.795 |
| DNKC | 1357 (28.9%) | 590 (29.4%) |  |
| KSCC | 1643 (35.0%) | 686 (34.1%) |  |
| Others | 1028 (21.9%) | 432 (21.5%) |  |
| UNKC | 671 (14.3%) | 302 (15.0%) |  |
| Tumor Site: |  |  | 0.045 |
| Anterior Wall | 40 (0.85%) | 26 (1.29%) |  |
| Lateral Wall | 412 (8.77%) | 141 (7.01%) |  |
| NOS | 3564 (75.8%) | 1534 (76.3%) |  |
| Overlapping Lesion | 176 (3.75%) | 93 (4.63%) |  |
| Posterior Wall | 461 (9.81%) | 200 (9.95%) |  |
| Superior Wall | 46 (0.98%) | 16 (0.80%) |  |
| Grade: |  |  | 0.911 |
| Grade I | 59 (1.26%) | 26 (1.29%) |  |
| Grade II | 384 (8.17%) | 160 (7.96%) |  |
| Grade III | 2426 (51.6%) | 1023 (50.9%) |  |
| Grade IV | 1830 (38.9%) | 801 (39.9%) |  |
| Tumor Size | 3.89 (1.57) | 3.94 (1.84) | 0.244 |
| T stage: |  |  | 0.976 |
| T1 | 1626 (34.6%) | 689 (34.3%) |  |
| T2 | 920 (19.6%) | 397 (19.8%) |  |
| T3 | 1028 (21.9%) | 448 (22.3%) |  |
| T4 | 1125 (23.9%) | 476 (23.7%) |  |
| N stage: |  |  | 0.278 |
| N0 | 997 (21.2%) | 402 (20.0%) |  |
| N1 | 1621 (34.5%) | 705 (35.1%) |  |
| N2 | 1475 (31.4%) | 666 (33.1%) |  |
| N3 | 606 (12.9%) | 237 (11.8%) |  |

**Supplementary Table 1.** Comprehensive baseline information for DM-all cohort.

|  | **training** | **testing** | **p-value** |
| --- | --- | --- | --- |
|  | ***n=4014*** | ***n=1719*** |  |
| Metastasis: |  |  | 0.161 |
| No | 3565 (88.8%) | 1549 (90.1%) |  |
| Yes | 449 (11.2%) | 170 (9.89%) |  |
| Age | 53.2 (15.0) | 53.5 (15.4) | 0.502 |
| Gender: |  |  | 0.536 |
| Female | 1115 (27.8%) | 492 (28.6%) |  |
| Male | 2899 (72.2%) | 1227 (71.4%) |  |
| Race: |  |  | 0.315 |
| AA | 60 (1.49%) | 23 (1.34%) |  |
| AP | 1769 (44.1%) | 721 (41.9%) |  |
| Black | 454 (11.3%) | 224 (13.0%) |  |
| Hispanic | 365 (9.09%) | 164 (9.54%) |  |
| White | 1366 (34.0%) | 587 (34.1%) |  |
| Marital: |  |  | 0.289 |
| Partnered | 2490 (62.0%) | 1030 (59.9%) |  |
| Previously Partnered | 575 (14.3%) | 253 (14.7%) |  |
| Single | 949 (23.6%) | 436 (25.4%) |  |
| Histology: |  |  | 0.693 |
| DNKC | 1172 (29.2%) | 525 (30.5%) |  |
| KSCC | 1392 (34.7%) | 586 (34.1%) |  |
| Others | 834 (20.8%) | 359 (20.9%) |  |
| UNKC | 616 (15.3%) | 249 (14.5%) |  |
| Site: |  |  | 0.824 |
| Anterior Wall | 37 (0.92%) | 17 (0.99%) |  |
| Lateral Wall | 351 (8.74%) | 159 (9.25%) |  |
| NOS | 2973 (74.1%) | 1286 (74.8%) |  |
| Overlapping Lesion | 181 (4.51%) | 68 (3.96%) |  |
| Posterior Wall | 429 (10.7%) | 174 (10.1%) |  |
| Superior Wall | 43 (1.07%) | 15 (0.87%) |  |
| T stage: |  |  | 0.413 |
| T1 | 1365 (34.0%) | 602 (35.0%) |  |
| T2 | 814 (20.3%) | 351 (20.4%) |  |
| T3 | 906 (22.6%) | 354 (20.6%) |  |
| T4 | 929 (23.1%) | 412 (24.0%) |  |
| N stage: |  |  | 0.713 |
| N0 | 821 (20.5%) | 369 (21.5%) |  |
| N1 | 1357 (33.8%) | 585 (34.0%) |  |
| N2 | 1322 (32.9%) | 542 (31.5%) |  |
| N3 | 514 (12.8%) | 223 (13.0%) |  |

**Supplementary Table 2.** Comprehensive baseline information for DM-slim cohort.

|  | **training** | **testing** | **p-value** |
| --- | --- | --- | --- |
|  | ***n=5823*** | ***n=2492*** |  |
| OS | 0.48 (0.50) | 0.48 (0.50) | 0.618 |
| OS Time | 5.66 (5.51) | 5.67 (5.47) | 0.923 |
| Age | 53.8 (15.3) | 52.9 (14.8) | 0.009 |
| Gender: |  |  | 0.250 |
| Female | 1618 (27.8%) | 724 (29.1%) |  |
| Male | 4205 (72.2%) | 1768 (70.9%) |  |
| Race: |  |  | 0.839 |
| AA | 87 (1.49%) | 41 (1.65%) |  |
| AP | 2552 (43.8%) | 1062 (42.6%) |  |
| Black | 647 (11.1%) | 290 (11.6%) |  |
| Hispanic | 519 (8.91%) | 228 (9.15%) |  |
| White | 2018 (34.7%) | 871 (35.0%) |  |
| Marital: |  |  | 0.782 |
| Partnered | 3626 (62.3%) | 1572 (63.1%) |  |
| Previously Partnered | 864 (14.8%) | 362 (14.5%) |  |
| Single | 1333 (22.9%) | 558 (22.4%) |  |
| Histology: |  |  | 0.973 |
| DNKC | 1540 (26.4%) | 654 (26.2%) |  |
| KSCC | 2050 (35.2%) | 888 (35.6%) |  |
| Others | 1285 (22.1%) | 552 (22.2%) |  |
| UNKC | 948 (16.3%) | 398 (16.0%) |  |
| Site: |  |  | 0.224 |
| Anterior Wall | 69 (1.18%) | 18 (0.72%) |  |
| Lateral Wall | 458 (7.87%) | 207 (8.31%) |  |
| NOS | 4413 (75.8%) | 1889 (75.8%) |  |
| Overlapping Lesion | 255 (4.38%) | 111 (4.45%) |  |
| Posterior Wall | 560 (9.62%) | 248 (9.95%) |  |
| Superior Wall | 68 (1.17%) | 19 (0.76%) |  |
| Grade: |  |  | 0.355 |
| Grade I | 72 (1.24%) | 33 (1.32%) |  |
| Grade II | 524 (9.00%) | 253 (10.2%) |  |
| Grade III | 2904 (49.9%) | 1209 (48.5%) |  |
| Grade IV | 2323 (39.9%) | 997 (40.0%) |  |
| T stage: |  |  | 0.970 |
| T1 | 2152 (37.0%) | 925 (37.1%) |  |
| T2 | 1095 (18.8%) | 459 (18.4%) |  |
| T3 | 1247 (21.4%) | 531 (21.3%) |  |
| T4 | 1329 (22.8%) | 577 (23.2%) |  |
| N stage: |  |  | 0.518 |
| N0 | 1228 (21.1%) | 535 (21.5%) |  |
| N1 | 2188 (37.6%) | 896 (36.0%) |  |
| N2 | 1729 (29.7%) | 753 (30.2%) |  |
| N3 | 678 (11.6%) | 308 (12.4%) |  |
| M stage: |  |  | 0.392 |
| M0 | 5227 (89.8%) | 2253 (90.4%) |  |
| M1 | 596 (10.2%) | 239 (9.59%) |  |
| Tumor size | 3.87 (1.58) | 3.91 (1.75) | 0.313 |
| SurgeryPS: |  |  | 0.435 |
| Local Excision | 405 (6.96%) | 155 (6.22%) |  |
| No Surgery | 5296 (90.9%) | 2275 (91.3%) |  |
| Pharyngectomy | 80 (1.37%) | 42 (1.69%) |  |
| Surgery NOS | 42 (0.72%) | 20 (0.80%) |  |
| SurgeryLN: |  |  | 0.237 |
| Biopsy | 796 (13.7%) | 348 (14.0%) |  |
| Lymph Nodes Removed | 457 (7.85%) | 169 (6.78%) |  |
| None | 4570 (78.5%) | 1975 (79.3%) |  |
| Chemotherapy: |  |  | 0.931 |
| No/Unknown | 1217 (20.9%) | 518 (20.8%) |  |
| Yes | 4606 (79.1%) | 1974 (79.2%) |  |
| Radiation: |  |  | 0.803 |
| Beam Radiation | 4788 (82.2%) | 2039 (81.8%) |  |
| No/Unknown | 969 (16.6%) | 421 (16.9%) |  |
| Other Radiation | 66 (1.13%) | 32 (1.28%) |  |
| Time_to_treatment: |  |  | 0.851 |
| Intermediate | 2712 (46.6%) | 1152 (46.2%) |  |
| Long | 224 (3.85%) | 91 (3.65%) |  |
| Timely | 2887 (49.6%) | 1249 (50.1%) |  |

**Supplementary Table 3.** Comprehensive baseline information for OS-all cohort.

|  | **training** | **testing** | **p-value** |
| --- | --- | --- | --- |
|  | ***n=3605*** | ***n=1542*** |  |
| OS | 0.39 (0.49) | 0.40 (0.49) | 0.846 |
| OS Time | 5.27 (4.70) | 5.34 (4.65) | 0.615 |
| Age | 52.8 (15.0) | 53.0 (15.1) | 0.749 |
| Gender: |  |  | 0.781 |
| Female | 1023 (28.4%) | 431 (28.0%) |  |
| Male | 2582 (71.6%) | 1111 (72.0%) |  |
| Race: |  |  | 0.672 |
| AA | 51 (1.41%) | 27 (1.75%) |  |
| AP | 1566 (43.4%) | 683 (44.3%) |  |
| Black | 429 (11.9%) | 180 (11.7%) |  |
| Hispanic | 332 (9.21%) | 152 (9.86%) |  |
| White | 1227 (34.0%) | 500 (32.4%) |  |
| Marital: |  |  | 0.302 |
| Partnered | 2244 (62.2%) | 951 (61.7%) |  |
| Previously Partnered | 486 (13.5%) | 232 (15.0%) |  |
| Single | 875 (24.3%) | 359 (23.3%) |  |
| Histology: |  |  | 0.942 |
| DNKC | 1092 (30.3%) | 465 (30.2%) |  |
| KSCC | 1207 (33.5%) | 526 (34.1%) |  |
| Others | 749 (20.8%) | 310 (20.1%) |  |
| UNKC | 557 (15.5%) | 241 (15.6%) |  |
| Site: |  |  | 0.126 |
| Anterior Wall | 30 (0.83%) | 19 (1.23%) |  |
| Lateral Wall | 348 (9.65%) | 116 (7.52%) |  |
| NOS | 2627 (72.9%) | 1162 (75.4%) |  |
| Overlapping Lesion | 166 (4.60%) | 66 (4.28%) |  |
| Posterior Wall | 397 (11.0%) | 163 (10.6%) |  |
| Superior Wall | 37 (1.03%) | 16 (1.04%) |  |
| T stage: |  |  | 0.103 |
| T1 | 1269 (35.2%) | 511 (33.1%) |  |
| T2 | 723 (20.1%) | 334 (21.7%) |  |
| T3 | 799 (22.2%) | 316 (20.5%) |  |
| T4 | 814 (22.6%) | 381 (24.7%) |  |
| N stage: |  |  | 0.634 |
| N0 | 691 (19.2%) | 313 (20.3%) |  |
| N1 | 1250 (34.7%) | 520 (33.7%) |  |
| N2 | 1185 (32.9%) | 517 (33.5%) |  |
| N3 | 479 (13.3%) | 192 (12.5%) |  |
| M stage: |  |  | 0.390 |
| M0 | 3229 (89.6%) | 1368 (88.7%) |  |
| M1 | 376 (10.4%) | 174 (11.3%) |  |
| SurgeryPS: |  |  | 0.271 |
| Local Excision | 263 (7.30%) | 109 (7.07%) |  |
| No Surgery | 3269 (90.7%) | 1397 (90.6%) |  |
| Pharyngectomy | 49 (1.36%) | 30 (1.95%) |  |
| Surgery NOS | 24 (0.67%) | 6 (0.39%) |  |
| SurgeryLN: |  |  | 0.218 |
| Biopsy | 632 (17.5%) | 243 (15.8%) |  |
| Lymph Nodes Removed | 281 (7.79%) | 133 (8.63%) |  |
| None | 2692 (74.7%) | 1166 (75.6%) |  |
| Chemotherapy: |  |  | 0.098 |
| No/Unknown | 392 (10.9%) | 193 (12.5%) |  |
| Yes | 3213 (89.1%) | 1349 (87.5%) |  |
| Radiation: |  |  | 0.086 |
| Beam Radiation | 3301 (91.6%) | 1389 (90.1%) |  |
| No/Unknown | 275 (7.63%) | 132 (8.56%) |  |
| Other Radiation | 29 (0.80%) | 21 (1.36%) |  |
| Time_to_treatment: |  |  | 0.601 |
| Intermediate | 1661 (46.1%) | 727 (47.1%) |  |
| Long | 154 (4.27%) | 58 (3.76%) |  |
| Timely | 1790 (49.7%) | 757 (49.1%) |  |

**Supplementary Table 4.** Comprehensive baseline information for OS-slim cohort.

|  | **training** | **testing** | **p-value** |
| --- | --- | --- | --- |
|  | ***n=5732*** | ***n=2454*** |  |
| CSS | 0.37 (0.48) | 0.38 (0.48) | 0.531 |
| CSS Time | 5.70 (5.53) | 5.58 (5.45) | 0.355 |
| Age | 53.4 (15.3) | 53.7 (14.9) | 0.402 |
| Gender: |  |  | 0.353 |
| Female | 1599 (27.9%) | 710 (28.9%) |  |
| Male | 4133 (72.1%) | 1744 (71.1%) |  |
| Race: |  |  | 0.917 |
| AA | 93 (1.62%) | 35 (1.43%) |  |
| AP | 2469 (43.1%) | 1064 (43.4%) |  |
| Black | 649 (11.3%) | 287 (11.7%) |  |
| Hispanic | 505 (8.81%) | 221 (9.01%) |  |
| White | 2016 (35.2%) | 847 (34.5%) |  |
| Marital: |  |  | 0.776 |
| Partnered | 3587 (62.6%) | 1528 (62.3%) |  |
| Previously Partnered | 830 (14.5%) | 370 (15.1%) |  |
| Single | 1315 (22.9%) | 556 (22.7%) |  |
| Histology: |  |  | 0.604 |
| DNKC | 1539 (26.8%) | 624 (25.4%) |  |
| KSCC | 2015 (35.2%) | 886 (36.1%) |  |
| Others | 1255 (21.9%) | 544 (22.2%) |  |
| UNKC | 923 (16.1%) | 400 (16.3%) |  |
| Site: |  |  | 0.144 |
| Anterior Wall | 57 (0.99%) | 27 (1.10%) |  |
| Lateral Wall | 467 (8.15%) | 191 (7.78%) |  |
| NOS | 4344 (75.8%) | 1860 (75.8%) |  |
| Overlapping Lesion | 267 (4.66%) | 89 (3.63%) |  |
| Posterior Wall | 535 (9.33%) | 263 (10.7%) |  |
| Superior Wall | 62 (1.08%) | 24 (0.98%) |  |
| Grade: |  |  | 0.429 |
| Grade I | 75 (1.31%) | 33 (1.34%) |  |
| Grade II | 537 (9.37%) | 223 (9.09%) |  |
| Grade III | 2805 (48.9%) | 1249 (50.9%) |  |
| Grade IV | 2315 (40.4%) | 949 (38.7%) |  |
| T stage: |  |  | 0.075 |
| T1 | 2206 (38.5%) | 883 (36.0%) |  |
| T2 | 1048 (18.3%) | 460 (18.7%) |  |
| T3 | 1183 (20.6%) | 560 (22.8%) |  |
| T4 | 1295 (22.6%) | 551 (22.5%) |  |
| N stage: |  |  | 0.537 |
| N0 | 1219 (21.3%) | 503 (20.5%) |  |
| N1 | 2112 (36.8%) | 916 (37.3%) |  |
| N2 | 1718 (30.0%) | 762 (31.1%) |  |
| N3 | 683 (11.9%) | 273 (11.1%) |  |
| M stage: |  |  | 0.011 |
| M0 | 5198 (90.7%) | 2180 (88.8%) |  |
| M1 | 534 (9.32%) | 274 (11.2%) |  |
| Tumor size | 3.86 (1.54) | 3.89 (1.87) | 0.535 |
| SurgeryPS: |  |  | 0.396 |
| Local Excision | 386 (6.73%) | 171 (6.97%) |  |
| No Surgery | 5209 (90.9%) | 2237 (91.2%) |  |
| Pharyngectomy | 88 (1.54%) | 33 (1.34%) |  |
| Surgery NOS | 49 (0.85%) | 13 (0.53%) |  |
| SurgeryLN: |  |  | 0.412 |
| Biopsy | 769 (13.4%) | 356 (14.5%) |  |
| Lymph Nodes Removed | 439 (7.66%) | 189 (7.70%) |  |
| None | 4524 (78.9%) | 1909 (77.8%) |  |
| Chemotherapy: |  |  | 0.907 |
| No/Unknown | 1190 (20.8%) | 513 (20.9%) |  |
| Yes | 4542 (79.2%) | 1941 (79.1%) |  |
| Radiation: |  |  | 0.577 |
| Beam Radiation | 4727 (82.5%) | 2000 (81.5%) |  |
| No/Unknown | 938 (16.4%) | 424 (17.3%) |  |
| Other Radiation | 67 (1.17%) | 30 (1.22%) |  |
| Time_to_treatment: |  |  | 0.492 |
| Intermediate | 2714 (47.3%) | 1127 (45.9%) |  |
| Long | 210 (3.66%) | 94 (3.83%) |  |
| Timely | 2808 (49.0%) | 1233 (50.2%) |  |

**Supplementary Table 5.** Comprehensive baseline information for CSS-all cohort.

|  | **training** | **testing** | **p-value** |
| --- | --- | --- | --- |
|  | ***N=3565*** | ***N=1526*** |  |
| CSS | 0.30 (0.46) | 0.31 (0.46) | 0.706 |
| CSS Time | 5.34 (4.70) | 5.23 (4.68) | 0.438 |
| Age | 52.9 (15.0) | 52.6 (15.1) | 0.618 |
| Gender: |  |  | 1.000 |
| Female | 1010 (28.3%) | 432 (28.3%) |  |
| Male | 2555 (71.7%) | 1094 (71.7%) |  |
| Race: |  |  | 0.462 |
| AA | 50 (1.40%) | 28 (1.83%) |  |
| AP | 1543 (43.3%) | 669 (43.8%) |  |
| Black | 441 (12.4%) | 168 (11.0%) |  |
| Hispanic | 326 (9.14%) | 150 (9.83%) |  |
| White | 1205 (33.8%) | 511 (33.5%) |  |
| Marital: |  |  | 0.806 |
| Partnered | 2219 (62.2%) | 935 (61.3%) |  |
| Previously Partnered | 496 (13.9%) | 217 (14.2%) |  |
| Single | 850 (23.8%) | 374 (24.5%) |  |
| Histology: |  |  | 0.963 |
| DNKC | 1073 (30.1%) | 469 (30.7%) |  |
| KSCC | 1206 (33.8%) | 514 (33.7%) |  |
| Others | 733 (20.6%) | 306 (20.1%) |  |
| UNKC | 553 (15.5%) | 237 (15.5%) |  |
| Site: |  |  | 0.827 |
| Anterior Wall | 35 (0.98%) | 13 (0.85%) |  |
| Lateral Wall | 312 (8.75%) | 148 (9.70%) |  |
| NOS | 2637 (74.0%) | 1110 (72.7%) |  |
| Overlapping Lesion | 162 (4.54%) | 66 (4.33%) |  |
| Posterior Wall | 381 (10.7%) | 174 (11.4%) |  |
| Superior Wall | 38 (1.07%) | 15 (0.98%) |  |
| T stage: |  |  | 0.131 |
| T1 | 1245 (34.9%) | 521 (34.1%) |  |
| T2 | 700 (19.6%) | 342 (22.4%) |  |
| T3 | 778 (21.8%) | 329 (21.6%) |  |
| T4 | 842 (23.6%) | 334 (21.9%) |  |
| N stage: |  |  | 0.752 |
| N0 | 709 (19.9%) | 284 (18.6%) |  |
| N1 | 1223 (34.3%) | 526 (34.5%) |  |
| N2 | 1172 (32.9%) | 512 (33.6%) |  |
| N3 | 461 (12.9%) | 204 (13.4%) |  |
| M stage: |  |  | 0.157 |
| M0 | 3203 (89.8%) | 1350 (88.5%) |  |
| M1 | 362 (10.2%) | 176 (11.5%) |  |
| SurgeryPS: |  |  | 0.550 |
| Local Excision | 269 (7.55%) | 101 (6.62%) |  |
| No Surgery | 3221 (90.4%) | 1392 (91.2%) |  |
| Pharyngectomy | 56 (1.57%) | 22 (1.44%) |  |
| Surgery NOS | 19 (0.53%) | 11 (0.72%) |  |
| SurgeryLN: |  |  | 0.296 |
| Biopsy | 586 (16.4%) | 278 (18.2%) |  |
| Lymph Nodes Removed | 288 (8.08%) | 123 (8.06%) |  |
| None | 2691 (75.5%) | 1125 (73.7%) |  |
| Chemotherapy: |  |  | 0.204 |
| No/Unknown | 417 (11.7%) | 159 (10.4%) |  |
| Yes | 3148 (88.3%) | 1367 (89.6%) |  |
| Radiation: |  |  | 0.252 |
| Beam Radiation | 3266 (91.6%) | 1379 (90.4%) |  |
| No/Unknown | 268 (7.52%) | 128 (8.39%) |  |
| Other Radiation | 31 (0.87%) | 19 (1.25%) |  |
| Time_to_treatment: |  |  | 0.590 |
| Intermediate | 1660 (46.6%) | 702 (46.0%) |  |
| Long | 152 (4.26%) | 57 (3.74%) |  |
| Timely | 1753 (49.2%) | 767 (50.3%) |  |

**Supplementary Table 6.** Comprehensive baseline information for CSS-slim cohort.
